# Focal to bilateral tonic–clonic seizures are associated with widespread network abnormality in temporal lobe epilepsy

**DOI:** 10.1111/epi.16819

**Published:** 2021-01-21

**Authors:** Nishant Sinha, Natalie Peternell, Gabrielle M. Schroeder, Jane de Tisi, Sjoerd B. Vos, Gavin P. Winston, John S. Duncan, Yujiang Wang, Peter N. Taylor

**Affiliations:** ^1^ Faculty of Medical Sciences Translational and Clinical Research Institute Newcastle University Newcastle Upon Tyne UK; ^2^ Computational Neuroscience, Neurology, and Psychiatry Lab Interdisciplinary Computing and Complex BioSystems Research Group School of Computing Newcastle University Newcastle Upon Tyne UK; ^3^ National Institute for Health Research University College London Hospitals Biomedical Research Centre University College London Queen Square Institute of Neurology London UK; ^4^ Centre for Medical Image Computing University College London London UK; ^5^ Neuroradiological Academic Unit University College London Queen Square Institute of Neurology University College London London UK; ^6^ Epilepsy Society MRI Unit Chalfont St Peter UK; ^7^ Division of Neurology Department of Medicine Queen’s University Kingston ON Canada

**Keywords:** connectome, diffusion MRI, drug‐resistant epilepsy, network abnormality, node abnormality, secondary generalized seizures

## Abstract

**Objective:**

Our objective was to identify whether the whole‐brain structural network alterations in patients with temporal lobe epilepsy (TLE) and focal to bilateral tonic–clonic seizures (FBTCS) differ from alterations in patients without FBTCS.

**Methods:**

We dichotomized a cohort of 83 drug‐resistant patients with TLE into those with and without FBTCS and compared each group to 29 healthy controls. For each subject, we used diffusion‐weighted magnetic resonance imaging to construct whole‐brain structural networks. First, we measured the extent of alterations by performing FBTCS‐negative (FBTCS−) versus control and FBTCS‐positive (FBTCS+) versus control comparisons, thereby delineating altered subnetworks of the whole‐brain structural network. Second, by standardizing each patient's networks using control networks, we measured the subject‐specific abnormality at every brain region in the network, thereby quantifying the spatial localization and the amount of abnormality in every patient.

**Results:**

Both FBTCS+ and FBTCS− patient groups had altered subnetworks with reduced fractional anisotropy and increased mean diffusivity compared to controls. The altered subnetwork in FBTCS+ patients was more widespread than in FBTCS− patients (441 connections altered at *t* > 3, *p* < .001 in FBTCS+ compared to 21 connections altered at *t* > 3, *p* = .01 in FBTCS−). Significantly greater abnormalities—aggregated over the entire brain network as well as assessed at the resolution of individual brain areas—were present in FBTCS+ patients (*p* < .001, *d* = .82, 95% confidence interval = .32–1.3). In contrast, the fewer abnormalities present in FBTCS− patients were mainly localized to the temporal and frontal areas.

**Significance:**

The whole‐brain structural network is altered to a greater and more widespread extent in patients with TLE and FBTCS. We suggest that these abnormal networks may serve as an underlying structural basis or consequence of the greater seizure spread observed in FBTCS.

**Key Points**

- Patients with drug‐resistant TLE and FBTCS have widespread abnormalities in whole‐brain structural networks spanning many interconnected regions
- Patient susceptibility to FBTCS can be measured from a node abnormality metric, which quantifies abnormality load patient‐specifically
- Regions in subcortical and parietal lobe—known to be implicated in FBTCS—have marked increase in node abnormality in TLE patients with FBTCS
- Abnormal networks may be an underlying structural basis or consequence of rapid seizure spread in FBTCS

## 1 INTRODUCTION

Focal to bilateral tonic–clonic seizures (FBTCS) of temporal lobe origin rapidly propagate to widespread brain areas, although with variable patient‐specific propagation patterns and clinical characteristics.^1^, ^2^ FBTCS are the most severe form of epileptic seizures that predispose patients to high risk of sudden unexpected death in epilepsy and seizure‐related injuries.^3^, ^4^, ^5^ FBTCS are an adverse prognostic factor for seizure freedom after temporal lobe resection.^6^, ^7^, ^8^ It remains unclear why temporal lobe seizures generalize in some patients but not in others.^9^, ^10^ It is crucial to identify factors that make some patients susceptible to FBTCS despite taking seizure‐suppressing medications.

Recognizing the need to quantify patient susceptibility to FBTCS, some studies have investigated a range of clinical factors to differentiate patients with and without FBTCS,^7^, ^11^ showing positive association with the presence of hippocampal sclerosis and negative association with ictal speech and pedal automatism.^7^ Many studies have suggested that impairments in specific brain regions support FBTCS, after finding disrupted structure and function in circuits mediated by thalamus and basal ganglia.^6^, ^12^, ^13^, ^14^, ^15^, ^16^, ^17^, ^18^ It has also been suggested that FBTCS have a different mechanism to primary generalized seizures, with more complex patient‐specific spread.^10^, ^19^, ^20^, ^21^, ^22^, ^23^ There is a need to investigate the full complexity of brain networks,^24^ beyond the canonical thalamocortical pathways,^12^ to delineate networks underlying FBTCS.

Patients with drug‐resistant temporal lobe epilepsy (TLE) are known to have structural abnormalities extending beyond the hippocampus and temporal lobe, forming a network of epileptogenic brain structures.^25^, ^26^, ^27^, ^28^ Greater whole‐brain structural network abnormalities predispose patients to persistent seizures after TLE surgery.^29^, ^30^ These abnormalities may be associated with the distributed nature of epileptic activity, the pathophysiology of seizure onset and propagation, and the response to medical and surgical therapies.^31^, ^32^ There is a dearth of information on how whole‐brain structural network abnormalities differ between patients with and without FBTCS.

In this study, we investigated the abnormalities in the whole‐brain structural network of TLE patients with and without FBTCS. We hypothesized that those with FBTCS would have more widespread abnormalities of white‐matter pathways, and to test this, we mapped the spatial arrangement of alterations in the whole‐brain structural network of TLE patients with and without FBTCS.^29^, ^30^, ^33^ We show that patients with localized spread of focal onset seizures have localized alterations in brain areas neighboring seizure onset, whereas patients with FBTCS have marked widespread abnormalities across the whole brain.

## 2 MATERIALS AND METHODS

### 2.1 Participants

We studied 83 patients with drug‐resistant unilateral TLE who were undergoing presurgical evaluation at the National Hospital of Neurology and Neurosurgery, London, United Kingdom, and 29 controls. The controls in this study were demographically matched and recruited from the local population through advertisements. All controls were screened as per an institute‐approved proforma to exclude medical history of neurological or psychiatric problems including drug/alcohol misuse. Clinical diagnosis of FBTCS was based on video‐electroencephalographic (EEG) telemetry, EEG, and historical data. Sixty patients had a history of temporal lobe seizures with FBTCS, and 23 patients did not. The three groups—TLE with FBTCS (FBTCS+), TLE without FBTCS (FBTCS−), and controls—were not significantly different in terms of age and gender. Patient details are provided in Table S1 and summarized in Table 1. Data were analyzed in this study under the approval of the Newcastle University Ethics Committee (reference number 1804/2020).

**TABLE 1:** Demographic and clinical data of patients

Groups/variables	FBTCS+	FBTCS−	Controls	Significance
Patients, *n*	60	23	29	
Sex, male/female	24/36	8/15	12/17	χ^2^_FBTCS±_ = .03, *p*_FBTCS±_ = .85 χ^2^_FBTCS+C_ = .01, *p*_FBTCS+C_ = .91 χ^2^_FBTCS‐C_ = .03, *p*_FBTCS‐C_ = .84
Age at dMRI, years, mean ± SD	38.8 ± 12.3	35.3 ± 8.4	38.0 ± 12.0	*p*_FBTCS±_ = .20 *p*_FBTCS+C_ = .70 *p*_FBTCS‐C_ = .67
Age at epilepsy onset, years, mean ± SD	15.2 ± 11.1	16.1 ± 9.9	N/A	*p*_FBTCS±_ = .51
Epilepsy duration, years, mean ± SD	24.7 ± 14.5	20.4 ± 12.4	N/A	*p*_FBTCS±_ = .26
Side, left/right	32/28	10/13	N/A	χ^2^_FBTCS±_ = .31, *p*_FBTCS±_ = .57
Hippocampal sclerosis, *n* (%)	36 (60%)	9 (39%)	N/A	χ^2^_FBTCS±_ = 2.14, *p*_FBTCS±_ = .14
Surgical outcome, seizure‐free/not seizure‐free during 2 years after surgery	28/32	12/11	N/A	χ^2^_FBTCS±_ = .04, *p*_FBTCS±_ = .84

Abbreviations: C, control; dMRI, diffusion‐weighted magnetic resonance imaging; FBTCS, focal to bilateral tonic–clonic seizures; N/A, not applicable.

### 2.2 Magnetic resonance imaging acquisition and data processing

Magnetic resonance imaging (MRI) data were acquired on a 3‐T GE Signa HDx scanner (General Electric). Standard imaging gradients with a maximum strength of 40 mTm^−1^ and slew rate of 150 Tm^−1^s^−1^ were used. All data were acquired using a body coil for transmission and eight‐channel phased array coil for reception. Standard clinical sequences were performed, including a coronal three‐dimensional (3D) T1‐weighted volumetric acquisition (matrix = 256 × 256 × 170, in‐plane resolution = .9375 × .9375 mm, slice thickness = 1.1 mm). For each participant, diffusion‐weighted MRI data were acquired using a cardiac‐triggered single‐shot spin‐echo planar imaging sequence with echo time = 73 ms. Sets of 60 contiguous 2.4‐mm‐thick axial slices were obtained covering the whole brain, with diffusion sensitizing gradients applied in each of 52 noncollinear directions (b‐value of 1200 mm^2^/s, δ = 21 ms, Δ = 29 ms using full gradient strength of 40 mTm^−1^) along with six non‐diffusion‐weighted scans. The gradient directions were calculated and ordered as described elsewhere.^34^ The field of view was 24 × 24 cm, and the acquisition matrix size was 96 × 96, zero filled to 128 × 128 during reconstruction, giving a reconstructed voxel size of 1.875 × 1.875 × 2.4 mm. The diffusion MRI acquisition time for a total of 3480 image slices was approximately 25 min (depending on subject heart rate).

Diffusion MRI data were first corrected for signal drift, then eddy current and movement artifacts were corrected using the FSL eddy_correct tool.^35^ The b‐vectors were then rotated appropriately using the “fdt‐rotate‐bvecs” tool as part of FSL. The diffusion data for each subject were registered and reconstructed to the standard ICBM‐152 space using the q‐space diffeomorphic reconstruction implemented in DSI studio.^36^ DSI studio fitted a diffusion tensor imaging (DTI) model on the diffusion MRI data. DTI model assumes that the velocity of water diffusion follows a 3D Gaussian distribution, and the tensor calculated is exactly the covariance matrix of the Gaussian. The reconstruction performed eigen analysis on the calculated tensor and exported the fractional anisotropy (FA) and mean diffusivity (MD) maps as implemented elsewhere.^37^

### 2.3 Construction of structural brain networks

For each participant, we constructed a structural brain network consisting of nodes and connections between the nodes as described previously.^38^ We defined 90 contiguous cortical and subcortical regions (nodes) from the automated anatomical labeling parcellation atlas as the nodes of the network.^39^ To identify the connectivity between the nodes, we applied a whole‐brain neuroanatomically verified atlas of the structural connectome comprising 500 000 streamlines obtained from deterministic fiber tracking.^40^ Nodes *i* and *j* were connected if a streamline ended in them. We weighted the connectivity across all streamlines that connect each pair of nodes by averaging the FA and MD values from the DTI measurements. Repeating this process for each pair of nodes *i* and *j* resulted in two (FA and MD) weighted connectivity matrices of size 90 × 90 per participant. The density of connections in the connectivity matrices across all participants was constant, which is a desirable graph property for cross‐sectional group analysis.^41^

### 2.4 Network alterations assessed from network‐based statistics

We applied network‐based statistics (NBS) to compare the structural brain network connectivity of (1) FBTCS+ patients versus controls and (2) FBTCS− patients versus controls. NBS is a widely used statistical approach for comparing network connections in two groups that identifies altered subnetworks.^33^

In NBS analysis, we first used *t*‐statistics to test each connection between nodes *i* and *j* of the connectivity matrix between patients and controls, resulting in a *t*‐score matrix. Second, from the *t*‐score matrix, we obtained a binary matrix, identifying those connections that showed a *t*‐value higher than a set *t*‐score threshold, and zeros otherwise. Third, from the binary matrix, we identified the size of the largest connected component, a subnetwork of nodes that showed alteration in patients. The size of the component is defined as the number of connections in the subnetwork, which we refer to as the extent of alteration. Fourth, we employed permutation testing to determine whether the size of altered subnetwork identified in patients occurs by chance. In permutation testing, we randomly permuted the group assignment of connectivity matrices between patients and controls 5000 times and computed the size of the largest connected component to obtain a null distribution. We then assigned a *p*‐value to the observed altered component size by computing the percentage of null distribution that exceeded the size of the observed altered subnetwork in patients. Fifth, we repeated the entire NBS analysis described above for *t*‐score thresholds ranging from .05 to 5 in steps of .05 to quantitatively verify the consistency of our findings independent of threshold choice.

### 2.5 Node alterations assessed from node abnormality

Node abnormality is a measure that identifies how the distribution of altered connections in a network may impact the nodes that they connect. Building on the emerging concepts of epilepsy being a disorder of abnormal nodes and networks,^29^, ^30^ we premised that nodes with more abnormal connections, relative to their total number of connections, are more likely to have altered function than a node with no or fewer abnormal connections. Notably there are two aspects to our premise: (1) identification of abnormal connections and (2) identification of abnormal nodes.

First, we identified the abnormal connections in each subject. For every connection present between node *i* and *j* in the structural network of a subject, we obtained a connection distribution from the equivalent connection between node *i* and *j* of the control networks. We calculated the *z*‐score of that connection as the number of standard deviations away from the mean, with the mean and standard deviation derived from the control distribution. For control subjects, we held out each control, computed the mean and standard deviation of each connection from the remaining controls, and computed the *z*‐scores of the control's edges relative to these distributions. By repeating this process for every connection, we standardized the FA‐weighted connectivity matrices against controls, obtaining a 90 × 90 *z*‐score connectivity matrix per subject. From the *z*‐score connectivity matrix of a subject, we computed a binary matrix with ones for those connections that showed a *z*‐value higher than a set *z*‐score threshold and zeros otherwise. The connections in this binary network are the abnormal connections with a high *z*‐score; we identified different levels of abnormal connections by setting *z*‐score threshold ranging from 1.5 to 3.5 in steps of .1.

Second, we identified abnormal nodes. For each node of the structural connectivity matrix, we calculated node abnormality, defined as the ratio of the number of abnormal connections to the total number of connections of the node. Specifically, we obtained the ratio between the node degrees of the binary network of abnormal connections derived above and the node degree of the nonbinarized *z*‐score network. From the node abnormality measure, we categorized each node as either normal or abnormal by applying a node abnormality threshold ranging from .01 to .20 in steps of .01. Thus, the node abnormality threshold identifies abnormal nodes by specifying the required proportion of abnormal connections in a node to render it abnormal.

By counting the total number of abnormal nodes in the whole‐brain network at each pair of *z*‐score and node abnormality thresholds, we derived the whole‐brain abnormality load. Likewise, for brain subnetworks connecting nodes within six brain areas—temporal (18 nodes), subcortical (14 nodes), frontal (26 nodes), parietal (14 nodes), occipital (12 nodes), and cingulate (six nodes)—we repeated the above analysis, determining the abnormality load per brain area per subject.

Finally, we compared the abnormality between FBTCS+ patients and controls and between FBTCS− patients and controls at three spatial resolutions: (1) the gross resolution, the abnormality load of the whole‐brain networks; (2) the coarse resolution, the abnormality load of six brain areas; and (3) the fine resolution, the node abnormality of individual abnormal nodes spread throughout the brain. In comparing patients and controls, we treated the abnormality in controls as the baseline measurement and applied estimation statistics to quantify abnormality in patients above and beyond that in controls.^42^ At the fine resolution, we also compared the node abnormality at each region of interest (ROI) directly between FBTCS+ and FBTCS− patient groups.

### 2.6 Statistical analysis and data availability

We followed a case–control approach to evaluate whether there are more alterations in structural brain network of FBTCS+ patients compared to controls than FBTCS− patients compared to controls. We assessed the alterations by applying NBS and node abnormality approaches.

NBS is a nonparametric method available as a MATLAB toolbox. Statistical tests performed within NBS analysis were (1) one‐tailed *t*‐test to calculate *t*‐score matrices and (2) one‐tailed permutation test (5000 permutations) to assign a *p*‐value to the size of the abnormal subnetwork. We set the significance level at .05; that is, an altered subnetwork in NBS was reported only when *p* < .05.

In the node abnormality analysis, we identified a *z*‐score and node abnormality threshold pair that was the most discriminatory (highest effect size) between FBTCS+ and FBTCS− patients (Figure S4). For statistical quantification, we first applied nonparametric Kruskal–Wallis test to check the null hypothesis that abnormality load in control, FBTCS−, and FBTCS+ originates from the same distribution. We then applied pairwise estimation statistics reporting Cohen *d* score and *p*‐values from one‐tailed nonparametric Wilcoxon rank sum test. Estimation statistics uses a combination of effect sizes and confidence intervals to analyze data and interpret results; they are considered more informative than null hypothesis significance testing.^42^ We measured the effect size nonparametrically by computing area under receiver operating characteristic curve (AUROC). We computed 95% bootstrap confidence intervals of Cohen *d* and AUROC using a bias‐corrected and accelerated percentile method from 5000 bootstrap resamples with replacement.

We make available all the anonymized q‐space diffeomorphic reconstructed (QSDR) brain networks of 83 patients and 29 controls included in this study at https://doi.org/10.5281/zenodo.4432083.

## 3 RESULTS

**FIGURE 1:**
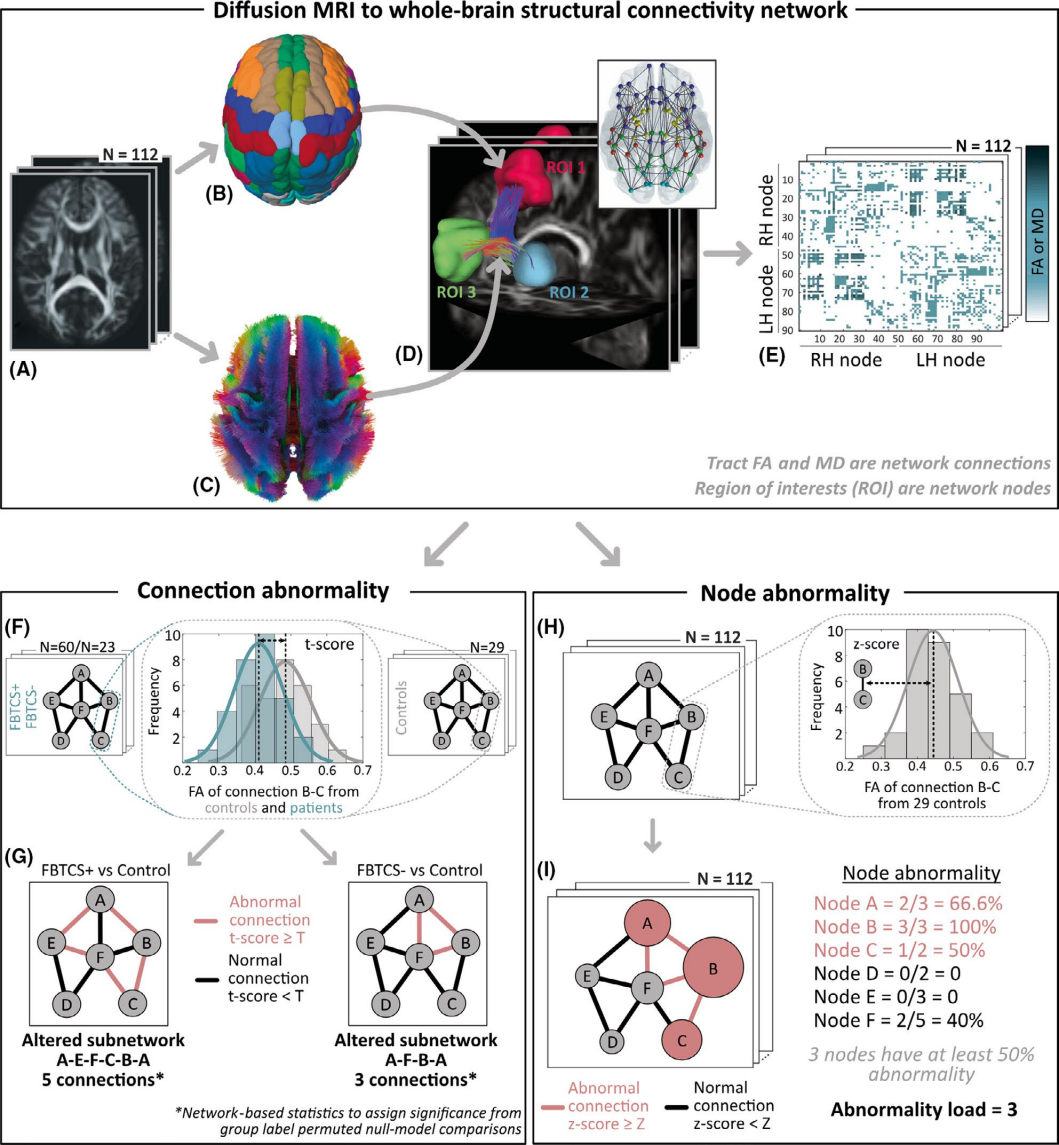
(A–E) *Overall approach: diffusion magnetic resonance imaging (MRI) to whole‐brain structural connectivity network*. (A) Diffusion MRI data from 112 participants (60 focal to bilateral tonic–clonic seizures [FBTCS]+ patients, 23 FBTCS− patients, and 29 controls) were QSDR reconstructed to align with the ICBM‐152 standard space. (B) Automated anatomical labeling parcellation atlas defined 90 cortical and subcortical regions of interest (ROIs). (C) The white‐matter streamlines constrained with neuroanatomical priors defined the connections between the ROIs. Streamlines are color‐coded as per the standard convention to indicate direction: red, left–right; green, anterior–posterior; blue, superior–inferior. (D) Three example ROIs with the streamlines ending in them as connections. By delineating connections between all pairs of ROIs, we derived a whole‐brain structural network for each participant (illustrated in the inset). (E) A network represented as a connectivity matrix with ROIs as nodes on the x‐ and y‐axes and connections encoded as the matrix element. We weighted the connections by averaging the fractional anisotropy (FA) or mean diffusivity (MD) values along the streamlines from diffusion tensor imaging measurements. Next, we assessed connection abnormality and node abnormality on these whole‐brain structural connectivity networks. For simplicity, we illustrate these concepts for a sample six‐node network. LH, left hemisphere; RH, right hemisphere. (F, G) *Connection abnormality*. (F) At every connection of the FBTCS+/FBTCS− patient groups and control group, we computed the *t*‐score as illustrated for a sample FA distribution of the connection B–C. (G) We defined abnormal (normal) connections as those above (below) a set *t*‐score threshold, T. By tracing the interconnected patterns of abnormal connections, we delineated an altered subnetwork, as shown in red, for FBTCS+ versus control and FBTCS− versus control comparisons. Network‐based statistics assessed the size of altered subnetwork from chance‐level occurrences in null models and assigned significance on the extent of alteration detected in the FBTCS+ and FBTCS− patient groups. (H, I) *Node abnormality*. (H) We computed the *z*‐score at each connection for every participant from the equivalent connection distribution in controls (illustrated for a sample connection (B–C). (I) We defined connections with *z*‐score higher or lower than a set threshold, Z, as abnormal (in red) or normal (in black). Node abnormality is the ratio of abnormal connections to the total number of connections in a node (illustrated by the size of the nodes). We identified abnormal nodes, shown in red, as those above a set node abnormality threshold, consequently quantifying abnormality load as the total number of abnormal nodes in the network.

Our main objective was to investigate whether the deviation in brain network structure from the normal range would be greater in patients with a history of FBTCS. We inferred the normal range of alterations from a control population and assessed the deviation in brain networks of patients in which focal seizures do not generalize (FBTCS−) and do generalize (FBTCS+). We hypothesized that most of the brain network structures in FBTCS− patients would be in the normal range, except some localized alterations in the temporal lobe. On the other hand, for FBTCS+ patients, we hypothesized widespread alterations in brain networks, given the rapid generalization of focal seizures to recruit widespread brain areas. Figure 1 summarizes our overall approach.

### 3.1 Widespread network alteration associated with secondary generalization of temporal lobe seizures

We investigated the alterations in brain networks of patients at the resolution of individual connections to identify the abnormal subnetwork and assess how large that subnetwork is in FBTCS+ and FBTCS− patients. We assumed that an interconnected configuration of altered connections—rather than altered connections in isolation or distributed randomly—would be the basis for focal onset seizures either remaining localized to a few areas or rapidly recruiting widespread areas. Therefore, we applied NBS to identify altered clusters of connections by comparing (1) FBTCS+ patients and controls and (2) FBTCS− patients and controls.

**FIGURE 2:**
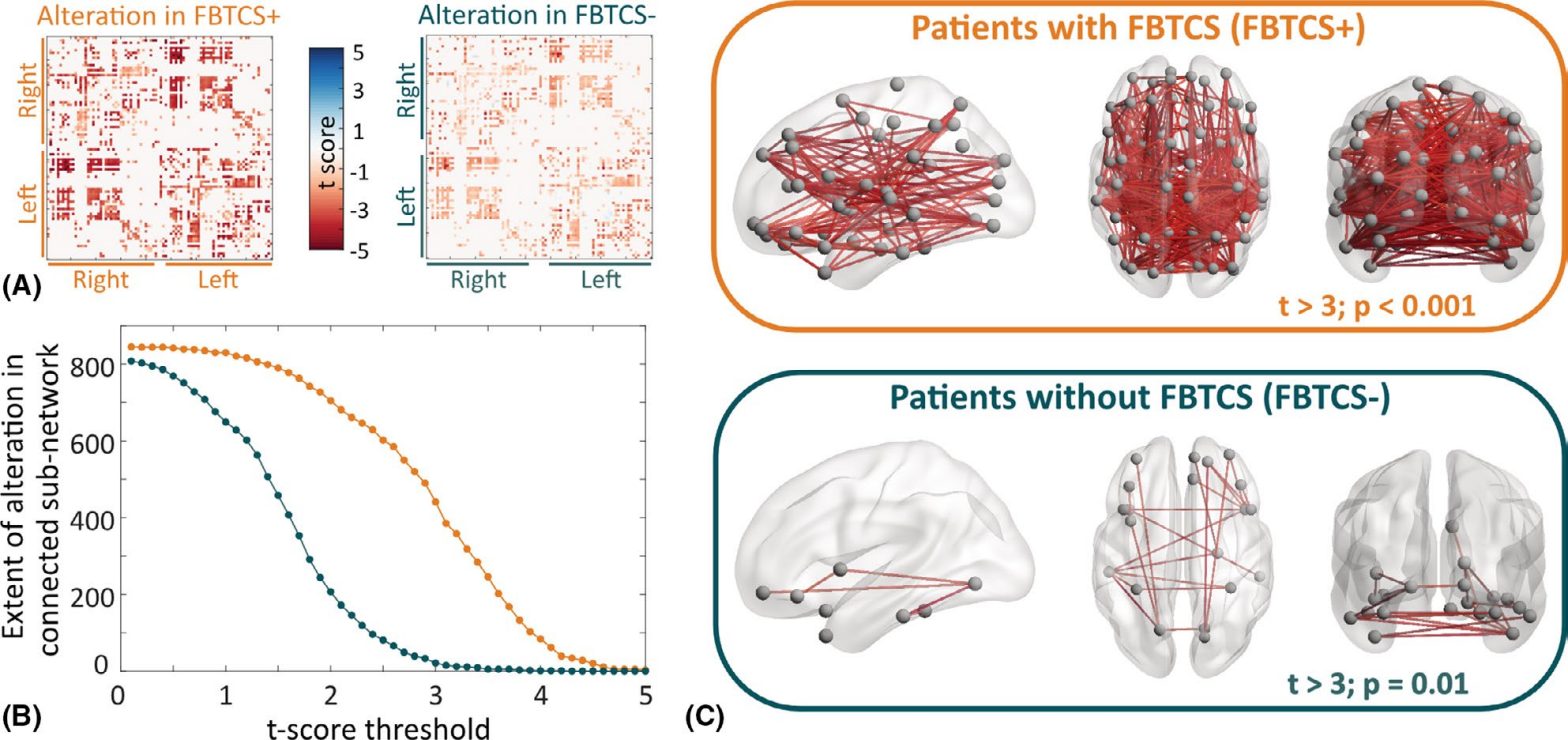
Widespread network alteration is associated with secondary generalization of temporal lobe seizures. We applied network‐based statistics (NBS) to compare fractional anisotropy (FA)‐weighted connectivity matrices of focal to bilateral tonic–clonic seizures (FBTCS)+ and FBTCS− patient groups with the control group. (A) Alteration of each connection quantified by *t*‐scores computed within the NBS analysis for FBTCS+ versus control group comparison on the left and FBTCS− versus control group comparison on the right. Negative (positive) *t*‐score indicates reduction (increase) in FA of patients compared to controls. We found that the lower negative *t*‐scores were widespread across many connections in FBTCS+ patients compared to FBTCS− patients. (B) Applying NBS analysis, we identified a significantly reduced subnetwork (connected component) at prespecified *t*‐score thresholds in FBTCS+ and FBTCS− patient groups compared to the control group. The number of edges contained in the altered subnetwork represents the extent of alteration. We detected that the FBTCS+ patients (in orange) have a higher extent of alteration than the FBTCS− patients (in teal) across all *t*‐score thresholds. (C) An example of a significantly reduced connected subnetwork in FBTCS+ and FBTCS− patients. FA at every edge of this subnetwork was reduced in patients with respect to controls with *t* > 3. While the altered subnetwork is widespread in the FBTCS+ patient group (upper panel), it is limited primarily to the regions in the temporal and frontal lobes in the FBTCS− patient group (lower panel)

Comparing FA‐weighted brain networks, we found that FBTCS+ patients have more widespread reductions in FA in many more connections than FBTCS− patients. Figure 2A illustrates these alterations using *t*‐statistics of the connections between regions. For a range of *t*‐score thresholds, we applied NBS delineating altered topological cluster, that is, the subnetwork of interconnected connections in which the *t*‐score of all connections is more than the specified threshold. Figure 2B illustrates the extent of alteration by plotting the number of connections in the altered subnetwork for FBTCS− versus control and FBTCS+ versus control comparisons. We found, across all *t*‐score thresholds, a larger altered subnetwork in FBTCS+ than FBTCS− patients. Figure 2C maps the spatial location of the altered connections at a sample *t*‐score threshold, *t* = 3. We found that in FBTCS− patients, the altered connections were localized in a subnetwork spanning temporal and frontal regions. However, in FBTCS+ patients, the subnetwork of altered connections was widespread, spanning many brain regions.

We observed similar results by applying the same analysis on (1) networks weighted by mean diffusivity in Figure S1 and (2) separately analyzing left TLE and right TLE patients in Figure S2.

In summary, we found that most of the connections in FBTCS− patients were in the normal range of healthy controls; the altered connections formed a subnetwork localizing primarily in the temporal and frontal areas. In contrast, in FBTCS+ patients, many connections deviated from the normal range of healthy controls, comprising a widespread subnetwork including brain regions distant from the temporal lobe.

### 3.2 Abnormality load and its spatial distribution associated with secondary generalization of temporal lobe seizures

Premising that the spatial arrangement of abnormal regions would relate to the site of seizure onset and spread, we mapped the abnormality of each region (or node) in the brain network. Specifically, for every subject we computed node abnormality—the ratio of abnormal connections to the total number of connections in a node—followed by the identification of abnormal nodes.^30^ We termed the total number of abnormal nodes at any given *z*‐score and node abnormality threshold pairs as the abnormality load (see Figure S3 and Materials and Methods for details). By comparing the abnormality load in controls, FBTCS+ patients, and FBTCS− patients, we determined the regions that had abnormalities outside the normal range of controls.

**FIGURE 3:**
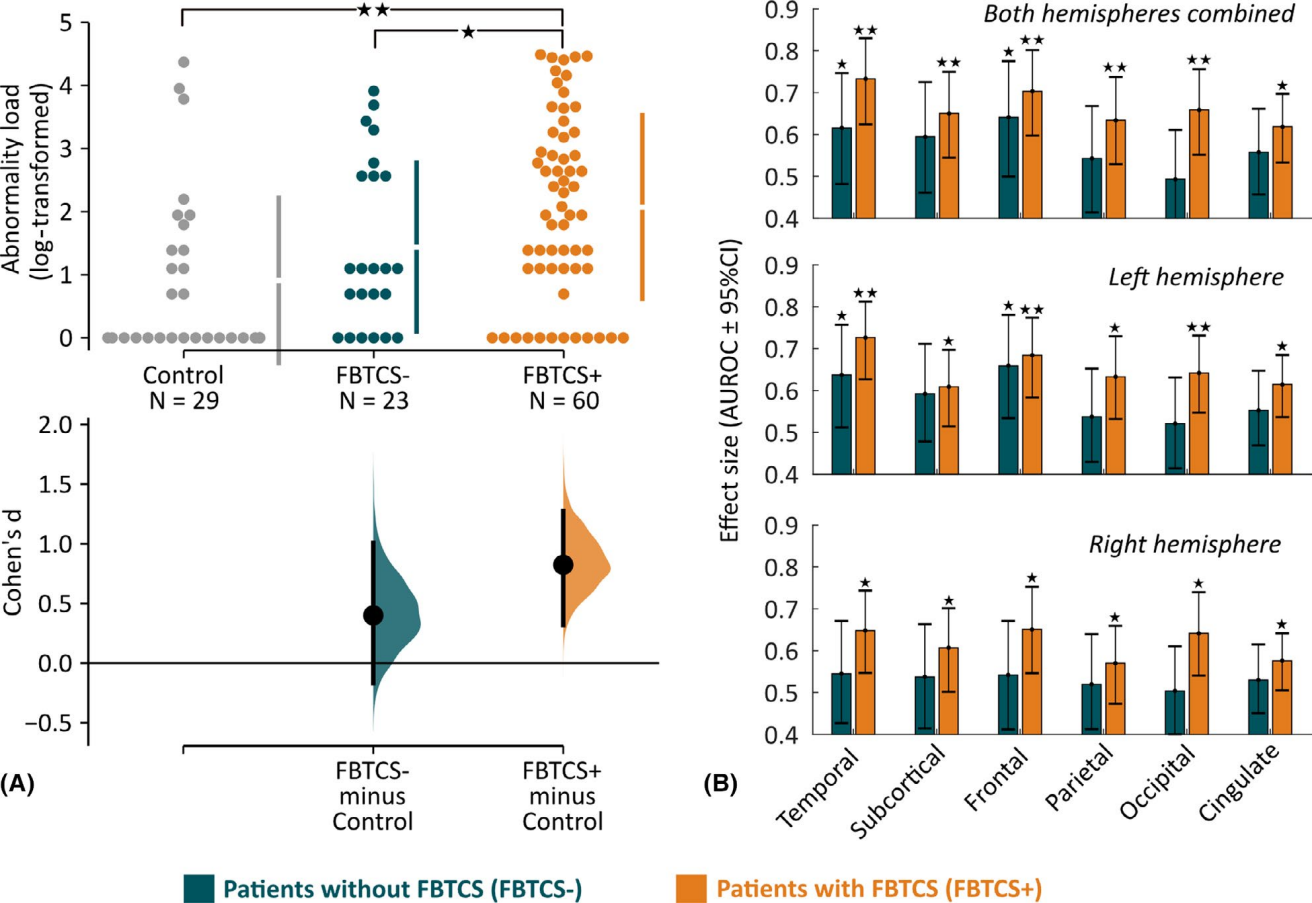
Abnormality load and its spatial distribution are associated with secondary generalization of temporal lobe seizures. (A) Abnormality load, that is, the total number of abnormal brain regions, is plotted on the estimation plot for the control, focal to bilateral tonic–clonic seizures (FBTCS)−, and FBTCS+ groups. Each dot represents a subject, the vertical lines represent the group mean with group standard deviation, and the lower panel shows the point estimate of Cohen *d* with 95% confidence interval (CI) from 5000 bootstrap resamples with replacement. We found that the abnormality load was significantly higher for FBTCS+ versus control group comparison as opposed to FBTCS− versus control group comparison. We also detected that the abnormality load in the FBTCS+ group was significantly higher than in the FBTCS− group. Statistical estimates: FBTCS− versus control: *p* = .04, *d* = .4, 95% CI = −.17 to 1; FBTCS+ versus control: *p* < .001, *d* = .82, 95% CI = .32–1.28; FBTCS+ versus FBTCS−: *p* = .03, *d* = .44, 95% CI = −.07 to .92. (B) At the resolution of individual lobes, the bar plot illustrates the effect size of abnormality load to discriminate between FBTCS− and control (in teal) and between FBTCS+ and control (in orange). We found that across all lobes, taken individually as left/right or combined, abnormality load in FBTCS+ was significantly higher than in the control group. In contrast, in the FBTCS− group only the abnormality load in temporal lobe (left and left‐right hemisphere combined) was significantly higher than in the control group. Two stars represent *p* < .005, and a single star represents .005 < *p* < .05. AUROC, area under receiver operating characteristic curve

First, at the entire brain network level, we found a significant difference in abnormality load by comparing controls, FBTCS− patients, and FBTCS+ patients (χ^2^ = 13.9, Kruskal–Wallis *p* < .001). The abnormality load in the FBTCS+ patient group was significantly higher than the FBTCS− patient and control groups (Figure 3A, upper panel). The estimation plot (Figure 3A, lower panel) shows that the effect size of abnormality load between FBTCS− and control is lower than FBTCS+ versus control. Statistical estimates are as follow: FBTCS− versus control: *p* = .04, *d* = .4, 95% confidence interval [CI] = −.17 to 1; FBTCS+ versus control: *p* < .001, *d* = .82, 95% CI = .32–1.28; FBTCS+ versus FBTCS−: *p* = .03, *d* = .44, 95% CI = −.07 to .92. Therefore, our results indicated that the whole‐brain abnormality load in the FBTCS− patient group was similar to the control group, and both were substantially lower compared to the FBTCS+ patient group.

Second, at the resolution of individual lobes/areas (Figure 3B), we found that abnormality load in FBTCS+ patients was substantially higher than controls across all lobes. In contrast, FBTCS− patients had substantially more abnormality load than controls only in the left temporal and left frontal lobes; other lobes, where seizures typically do not spread to, were not different from the baseline control level.

**FIGURE 4:**
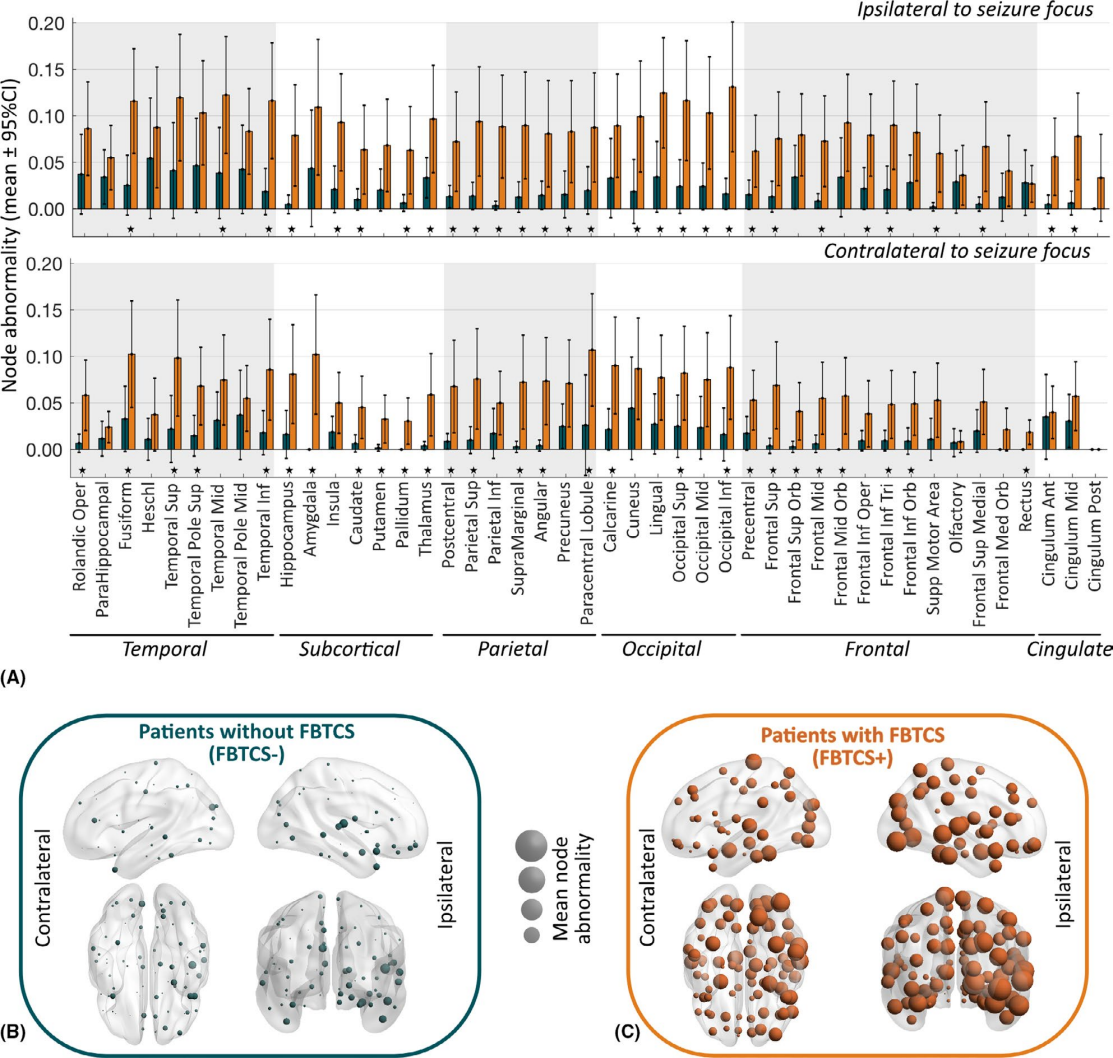
Node abnormality in regions ipsilateral and contralateral to seizure focus between patients with and without focal to bilateral tonic–clonic seizures (FBTCS). (A) At every region of interest (ROI) expressed as ipsilateral or contralateral to seizure focus, we computed the mean node abnormality with 95% confidence interval (CI) at *z*‐score > 2.5. Node abnormality in the ipsilateral hemisphere was higher than in the contralateral hemisphere. The FBTCS+ patient group (in orange) had greater node abnormality than the FBTCS− patient group (in teal) across all ROIs. Specific ROIs with significantly higher node abnormality in the FBTCS+ group than in the FBTCS− group are highlighted by stars representing *p* < .05 after Benjamini–Hochberg false discovery rate correction for multiple comparisons. Lobewise occurrence of ROIs with significantly higher node abnormality in the FBTCS+ group was as follows: temporal 8/18 (44%), subcortical 11/14 (78%), parietal 12/14 (85%), occipital 8/12 (66%), frontal 15/26 (57%), cingulate 2/6 (33%). (B, C) Mean node abnormality is mapped for FBTCS− patients in B and FBTCS+ patients in C. The size of the nodes, shown by spheres, is scaled to their mean node abnormality value. We found that in both patient groups node abnormality is higher in the ipsilateral temporal lobe relative to the abnormality in the rest of the brain. High node abnormality was widespread in the FBTCS+ patient group, whereas in the FBTCS− patient group the abnormal nodes were localized mainly in the temporal and frontal areas

Third, at a finer spatial resolution of 90 parcellated regions, we compared the node abnormality of every node in FBTCS− versus control and FBTCS+ versus control. By flipping the ROIs between left and right hemisphere of the left TLE patients, we expressed each ROI as either ipsilateral or contralateral to seizure focus. The mean node abnormality in FBTCS+ patients was significantly higher than FBTCS− patients in 29 ipsilateral and 27 contralateral ROIs, with the highest prevalence in the ROIs belonging to subcortical and parietal areas (Figure 4A). Figure S4 shows consistency of these results across a range of *z*‐score thresholds. Figure 4B,C maps the node abnormality at each ROI for the FBTCS− and FBTCS+ patient groups; the size of ROIs corresponds proportionally to their mean node abnormality. Many nodes in FBTCS+ patients have abnormalities greater than controls; abnormal nodes in FBTCS− patients are mostly localized in the ipsilateral temporal and frontal lobes.

In summary, we found that the abnormal nodes are spatially correlated with the site of seizure onset and spread. Patients in the FBTCS− group displayed localized abnormalities mainly in the temporal and frontal lobes, whereas FBTCS+ patients displayed widespread abnormalities. On average, FBTCS+ patients have significantly higher node abnormality than FBTCS− patients across many widespread ROIs, including subcortical and parietal areas.

## 4 DISCUSSION

We investigated whether widespread brain network abnormalities were present in patients with a history of FBTCS and drug‐resistant TLE. By comparing controls and patients with and without FBTCS, we mapped alterations in brain networks at the resolution of individual connections, nodes, lobes, and the whole brain. In patients without a history of FBTCS, abnormalities were localized mainly in temporal and frontal areas. In contrast, abnormalities were widespread and bilateral in patients with FBTCS. Regions in the subcortical and parietal lobes showed a marked increase in node abnormality in TLE patients with FBTCS. Abnormality load, a subject‐specific measure of whole‐brain abnormality, placed FBTCS patients at the higher end of the abnormality spectrum, followed by patients without FBTCS and then controls.

Alterations of white‐matter tracts, generally characterized by reduced anisotropy and increased diffusivity, are a feature of TLE.^26^ Here, we additionally showed higher and more widespread alterations in TLE patients with FBTCS. Pseudoprospective analysis (i.e., holding out a few patients as test cases, akin to new incoming patients) from cross‐validated machine learning models suggested the amount of abnormality expected to remain after surgery is an important factor determining seizure recurrence.^30^, ^43^ Other studies have shown an association between history of FBTCS and seizure outcome after TLE surgery.^6^, ^7^ Taken together, we suggest abnormality in whole‐brain structural connectivity may underpin both postsurgical seizure recurrence and presurgery FBTCS occurrence.

The pathophysiology of FBTCS is understood to involve disrupted network interactions between different brain areas. Local ictal discharges bilaterally propagate to brainstem motor areas via the corpus callosum to trigger the tonic–clonic phase.^20^, ^21^ Motor areas project excitatory activity to the thalamic nuclei and subcortical structures. From the thalamocortical projections, the excitatory seizure activity propagates to widespread areas after the inhibitory process fails at the basal ganglia.^12^, ^17^ Structural and functional abnormalities have been reported in these areas in patients with FBTCS.^12^, ^14^, ^15^, ^16^, ^20^ This hypothesis about the propagation model of FBTCS has primarily been supported by studies incorporating functional imaging modalities and/or T1‐weighted MRI. Surprisingly, only a few studies have utilized diffusion MRI to study FBTCS, limiting its application to thalamus‐associated fiber bundles.^14^, ^16^ We utilized diffusion MRI to study the whole‐brain structural network in FBTCS. Studying one of the largest patient cohorts at a single center, we found bilateral structural network abnormalities in regions belonging to the subcortical and parietal areas. These abnormal regions included the bilateral thalamus and motor areas, as described in the aforementioned propagation model of FBTCS. Therefore, our analysis provides complementary evidence from the diffusion MRI domain in support of the propagation model of FBTCS. In addition, our whole‐brain structural network analysis revealed abnormalities in other brain areas, thus suggesting that wider network disruption is present in individuals with FBTCS. Patients without FBTCS also have network disruption, but more localized. Although causality is difficult to infer, it is plausible that the recruitment of recurrent excitation pathways may have reinforced seizure generation and seizure propagation networks, thus leading to widespread abnormality in secondary generalized seizures versus localized abnormality in focal‐only seizures.^44^ Hence, we postulate abnormal neuroplasticity as the pathological mechanism underlying FBTCS.

Our novel application of the node abnormality metric allows the mapping of abnormalities on the whole‐brain structural network in a patient‐specific manner. Our analysis showed structural brain network abnormalities are greater and more widespread in patients with FBTCS. The node abnormality method reconciles the widespread alterations into a single patient‐specific metric: the abnormality load that was associated with FBTCS. Although we detected abnormalities in specific regions known to be involved in the pathophysiology of FBTCS, we also found abnormalities outside of those regions. Our results therefore suggest that although thalamocortical pathways, including the regions in subcortical and parietal lobes, are altered in FBTCS and might be important in understanding population‐level mechanisms of FBTCS, there may not be any one region that is specific for stratifying patients on the FBTCS spectrum. Instead, it is the total number of abnormal regions—a patient‐specific property—that is associated with the predisposition to FBTCS.

Our findings have implications for both existing and new treatments. Individual patients have different susceptibility to FBTCS, and there is a high clinical value in identifying who is at a higher risk of FBTCS. Although identifying mean group differences pertaining to a disease is crucial to develop mechanistic insights, personalized medicine requires quantifying patient‐specific heterogeneities.^45^ Metrics such as node abnormality,^30^ network abnormality,^29^ and deviation score^13^, ^17^ can quantify patient‐specific heterogeneities, thus stratifying patients on a spectrum of disease severity rather than dichotomized groups. Multivariate combinations of clinical factors associated with FBTCS^7^, ^11^ with our proposed patient‐specific abnormality measure may be able to determine patient susceptibility to FBTCS. Identifying predisposition of patients to FBTCS may be particularly relevant in epilepsy monitoring units, where anti‐seizure drug tapering carries a risk of FBTCS.^46^ For neuromodulation therapies, regions with high abnormality in a patient might be hypothesized as choke points for terminating seizures.^47^ We propose exploring the usefulness of patient‐specific abnormality measures for personalized treatment options.

Our findings should be interpreted with some caveats. First, the case–control design of our study could not detangle the cause–effect mechanisms underlying abnormality. Widespread abnormalities in the whole‐brain structural network could be either the cause or the effect of FBTCS. A longitudinal study of patients with new onset epilepsy is best suited to address these questions. Second, we could not study the left TLE and right TLE patients separately with high statistical power due to fewer patients remaining in the FBTCS− group. However, this limitation is mitigated to some extent due to the balance between left and right TLE patients in the FBTCS+ and FBTCS− groups and partly addressed by our combined ipsilateral–contralateral abnormality analysis (Figure 4). Third, we focused only on identifying features that make a *patient susceptible* to FBTCS and not on predicting whether a given *seizure* in a patient would generalize or remain focal. Even in a patient who is susceptible to secondary generalized seizures, only some seizures may generalize; the importance of within‐patient seizure variability and seizure‐specific treatment has been underscored recently.^48^ Identifying features associated with secondary generalization of seizures is also important,^2^, ^49^, ^50^ and a future multimodal analysis combining whole‐brain structural and functional networks would allow identification of seizure spreading on abnormal structural network substrates.

In conclusion, we have shown using diffusion MRI that widespread brain network abnormalities are present in patients with FBTCS. Measuring the extent and amount of abnormality on a patient‐specific whole‐brain structural network is a likely indication of patient susceptibility to secondary generalized seizures. Determining the likelihood of patients to have FBTCS is clinically important, because it offers the opportunity to intervene with personalized treatments.

## CONFLICT OF INTEREST

None of the authors has any conflict of interest to disclose.

## Supporting information

Supplementary Material

Table S1
