# Supplementary material for: Focal to bilateral tonic–clonic seizures are associated with widespread network abnormality in temporal lobe epilepsy
Source: Epilepsia. 2021 Jan 21;62(3):729–41. doi: 10.1111/epi.16819 (PMC8600951; doi:10.1111/epi.16819)
Supplement: Supplementary file 1 — Supplementary Material [file EPI-62-729-s001.docx]

# **Supplementary: Focal to bilateral tonic-clonic seizures are associated with widespread network abnormality in temporal lobe epilepsy**

## Nishant Sinha^1,2*^, Natalie Peternell^2^, Gabrielle M. Schroeder^2^, Jane de Tisi^3^, Sjoerd B. Vos^3,4,5^, Gavin P. Winston^3,6,7^, John S. Duncan^3,6^, Yujiang Wang^2,3^, Peter N. Taylor^2,3*^

### ^1^Translational and Clinical Research Institute, Faculty of Medical Sciences, Newcastle University, Newcastle upon Tyne, United Kingdom

### ^2^Computational Neuroscience, Neurology, and Psychiatry Lab, ICOS Group, School of Computing, Newcastle University, Newcastle upon Tyne, United Kingdom

### ^3^NIHR University College London Hospitals Biomedical Research Centre, UCL Queen Square Institute of Neurology, London, United Kingdom

### ^4^Centre for Medical Image Computing, University College London, London, United Kingdom

### ^5^Neuroradiological Academic Unit, UCL Queen Square Institute of Neurology, University College London, London, United Kingdom

### ^6^Epilepsy Society MRI Unit, Chalfont St Peter, United Kingdom

### ^7^Department of Medicine, Division of Neurology, Queen’s University, Kingston, Canada

^*^Corresponding author: [nishant.sinha89@gmail.com](mailto:nishant.sinha89@gmail.com) (NS); peter.taylor@newcastle.ac.uk (PNT)

Address: Urban Sciences Building, 1 Science Square, Newcastle Upon Tyne, NE4 5TG, Tyne and Wear, UK

**
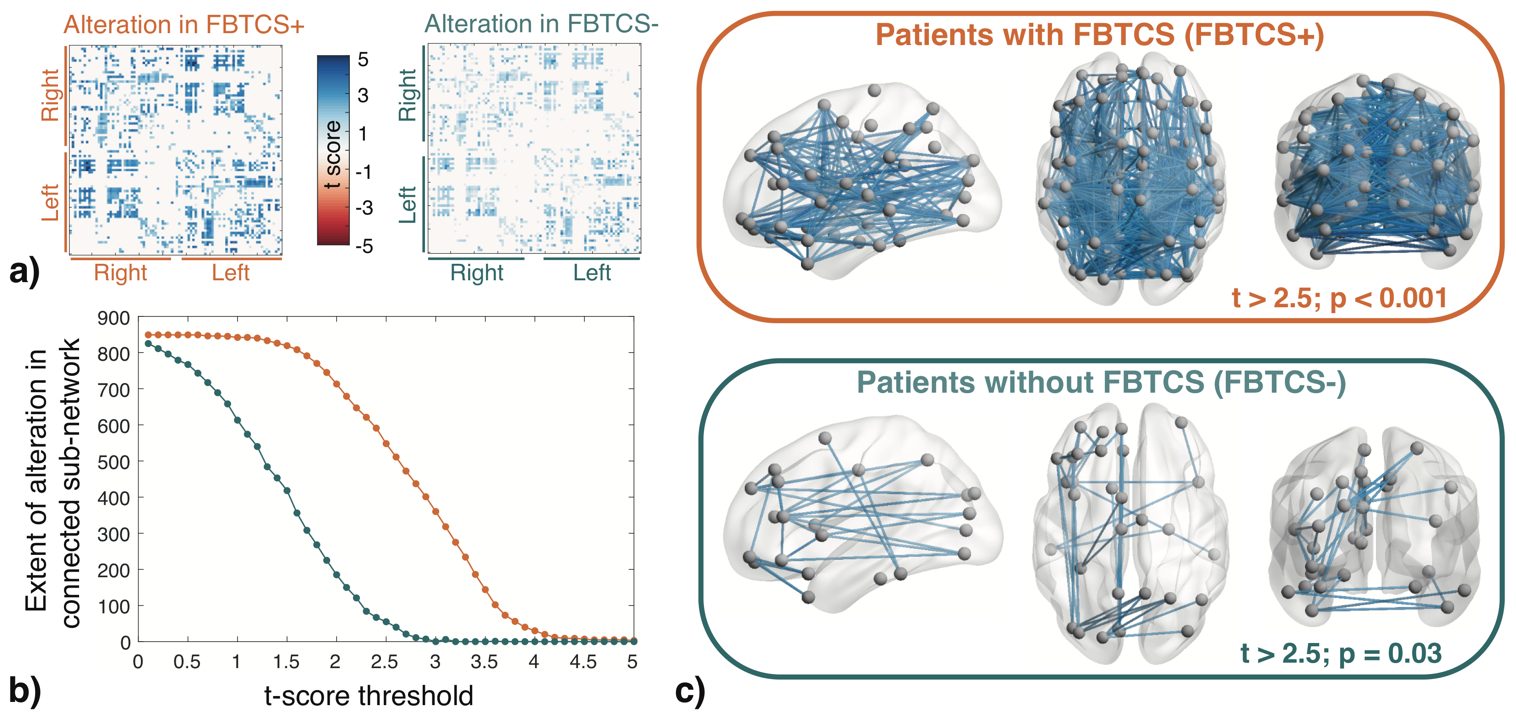
**

**Figure S1: Widespread network alterations associate with secondary generalisation of temporal lobe seizures. This figure is equivalent of Figure 2 for mean diffusivity weighted whole-brain structural networks.** We applied NBS to compare MD weighted connectivity matrices of FBTCS+ and FBTCS- patient groups with the control group. Panel **a)** illustrate alteration of each connection quantified by t-scores computed within the NBS analysis for FBTCS+ vs. control group comparison on the left and FBTCS- vs. control group comparison on the right. Positive (negative) t-score indicates increase (decrease) in MD of patients compared to controls. We found that the higher positive t-scores were widespread across many connections in FBTCS+ patients compared to FBTCS- patients. **b)** Applying NBS analysis, we identified significantly increased subnetwork (connected component) at pre-specified t-score thresholds in FBTCS+ and FBTCS- patient groups compared to control group. The number of edges contained in the altered subnetwork represents the extent of alteration. We detected that the FBTCS+ patients (in orange) have higher extent of alteration than the FBTCS- patients (in teal) across all t-score thresholds. **c)** An example of significantly increased connected subnetwork in FBTCS+ and FBTCS- patients; MD at every edge of this subnetwork was reduced in patient with respect to controls with t > 2.5. While the altered subnetwork is widespread in FBTCS+ patient group (upper panel), it is limited primarily to the regions in the temporal, frontal, and occipital lobes in FBTCS- patient group (lower panel).

**
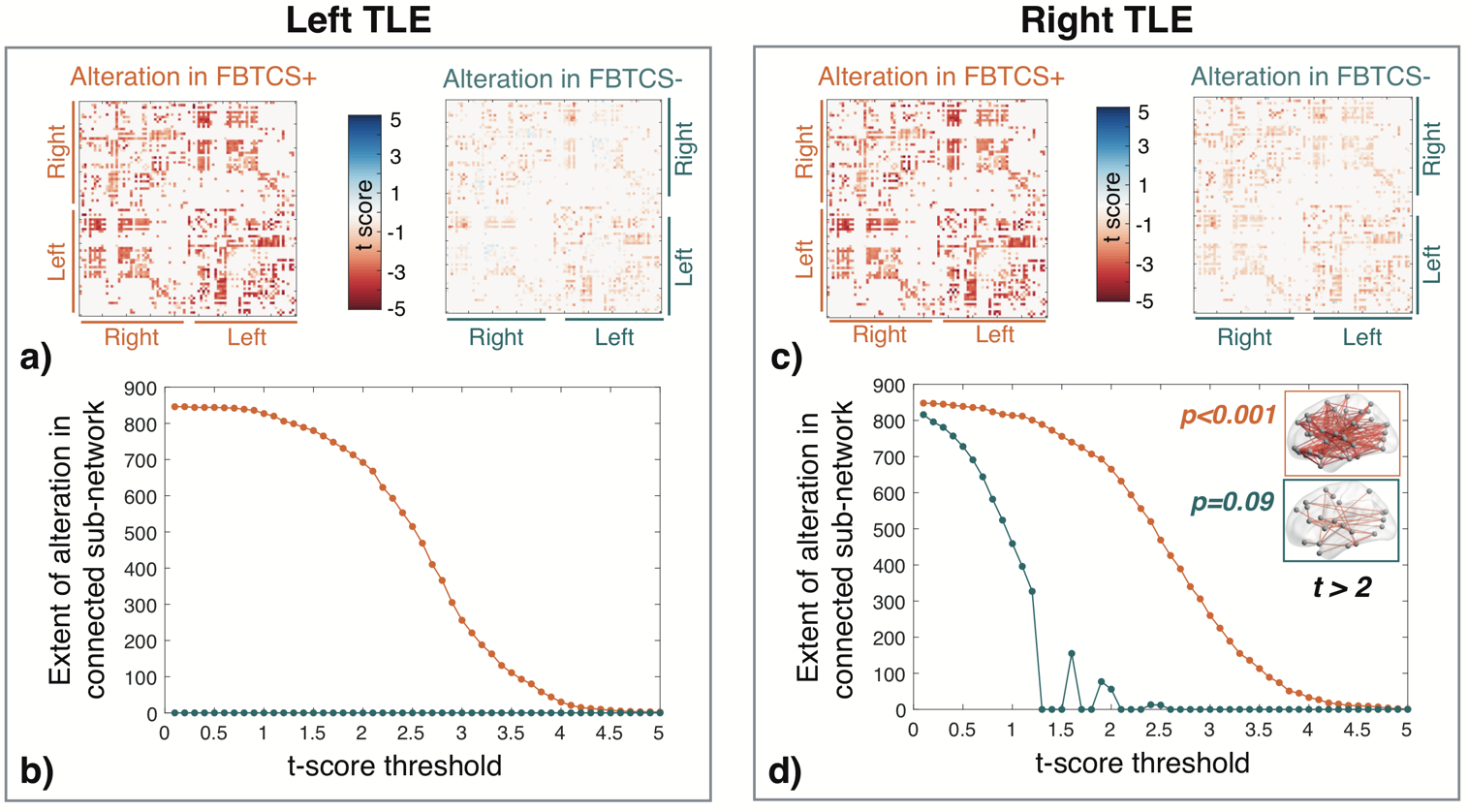
**

**Figure S2: Widespread network alterations associate with secondary generalisation of temporal lobe seizures even after separately analysing the left and right TLE patients.** We repeated the NBS analysis after separating the left and right TLE patients. For the left TLE analysis shown in the left panel **(a-b)** 32 patients had a history of FBTCS (FBTCS+) and 10 patients had focal-only seizures (FBTCS-). For the right TLE analysis shown in the right panel **(c-d)** 28 patients had a history of FBTCS (FBTCS+) and 13 patients had focal-only seizures (FBTCS-). As shown in panel **a)** and **c),** we found higher positive t-scores were widespread across many connections in FBTCS+ patients compared to FBTCS- patients in both left and right TLE analyses. **b-d)** Applying NBS analysis, we detected that FBTCS+ patients (in orange) have higher extent of alteration than FBTCS- patients (in teal) across all t-score thresholds in both left and right TLE patients analysed separately. Due to the reduced statistical power in FBTCS- patient group, we did not detect any subnetwork that was significantly reduced at p<0.05. Examples of significantly reduced connected subnetwork in FBTCS+ and FBTCS- patients are shown in the inset of panel **d)** for t > 2. These findings shown here for separate left and right TLE analysis are consistent with our combined left-right TLE analysis shown in Figure 2.


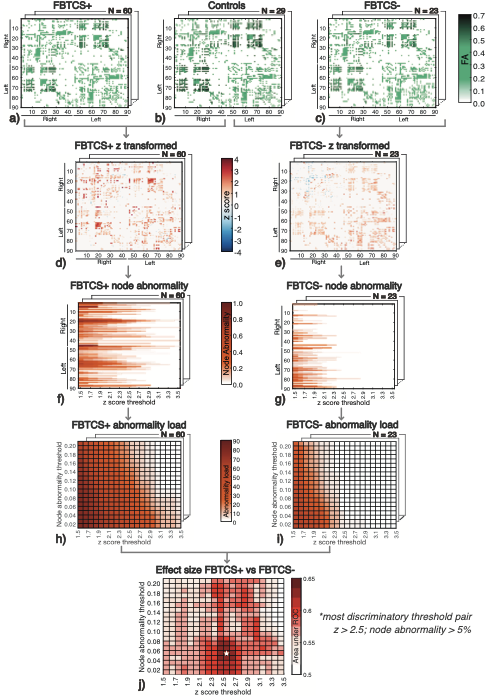


**Figure S3: Computation of abnormality load and identification of most discriminatory threshold pairs for abnormality load computation. a-c)** We standardised (z-score) the FA weighted connectivity matrices for 60 FBTCS+ patients and 23 FBTCS- at each connection with respect to the corresponding connection distribution obtained from 29 controls. Mathematically, this can be represented as $\left( \frac{\mu_{ij}^{controls}- k_{ij}^{patient}}{\sigma_{ij}^{controls}} \right)$, where $k_{ij}^{patient}$ is the FA of a connection between node *i* and *j* in patients, $\mu_{ij}^{controls}$ is the mean FA of connection between node *i* and *j* across controls, and $\sigma_{ij}^{controls}$ is the standard deviation of FA of connection between node *i* and *j* in controls. Panel **d)** shows the z-transformed connectivity matrices for FBTCS+ patients and panel **e)** for FBTCS- patients. Note that majority of z scores in these standardised networks are positive, i.e. FA of connections in patients were abnormally reduced. **f-g)** We computed node abnormality at every node (shown on the y-axis) as the ratio of total number of abnormal connections to the total number of connections at that node. We defined abnormal connections as those above a set z-score threshold (shown on the x-axis) ranging from 1.5 to 3.5 in steps of 0.1. Panel **f)** shows the node abnormality for FBTCS+ patients and panel **g)** for the FBTCS- patients. **h-i)** We identified abnormal nodes as those with node abnormality above a set node abnormality threshold. Node abnormality thresholds (ranging from 0.01 to 0.20 in steps of 0.01) are shown on the y-axis corresponding to every z-score threshold shown on the x-axis. The range for z-score and node abnormality thresholds are empirically chosen. We restricted the maximum range for z-score at 3.5 and node abnormality at 0.2 because beyond these threshold values no to very few abnormal connections and nodes were detected in most patients. At every pair of node abnormality threshold and z-score threshold, we identified each node as normal (0) or abnormal (1). We defined abnormality load as the total number of abnormal nodes identified at each pair of thresholds. Abnormality load for FBTCS+ patients are shown in panel **h)** and FBTCS- patients are shown in panel **i)**. In panel **j)** we computed the discrimination between FBTCS+ and FBTCS- patients at every threshold pair. Area under the receiver operator characteristic curve, a non-parametric measure of effect size, is plotted at every threshold pair. While high effect size (shown in red clusters) occurs at many threshold pairs, the star marks the threshold pair with the highest discrimination between FBTCS+ and FBTCS- patients.

**
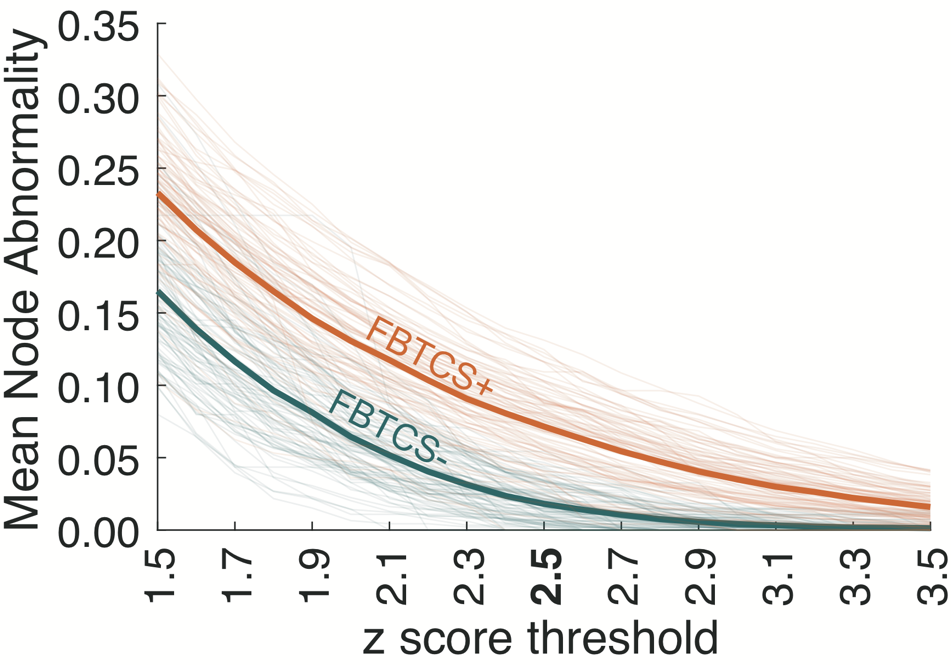
**

**Figure S4: Mean node abnormality of FBTCS+ patient group is higher than FBTCS- patient group across the range of z-score threshold.** Solid line plots the mean node abnormality across all ROI in FBTCS+ and FBTCS- patient groups at different z-score thresholds. Shaded lines show the mean node abnormality for each ROI in FBTCS+ and FBTCS- group. Equivalent expanded figure at z-score threshold of 2.5 is detailed in Figure 4 of the manuscript.
