# Supplementary material for: Focal to bilateral tonic–clonic seizures are associated with widespread network abnormality in temporal lobe epilepsy
Source: Epilepsia. 2021 Jan 21;62(3):729–41. doi: 10.1111/epi.16819 (PMC8600951; doi:10.1111/epi.16819)
Supplement: Supplementary file 2 — Table S1 [file EPI-62-729-s002.pdf]

Table S1

Subject	Sex	Age at dMRI (years)	Age at epilepsy onset (years)	Age at surgery (years)	Epilepsy duration (years)	History of FBTCS	Side of surgery	Hippocampal Sclerosis	ILAE outcome in 2 years	Surgical outcome
P1	F	35.7	23.0	36.6	13.6	N	R	N	2	NSF
P2	M	29.0	15.0	33.5	18.5	Y	R	N	4	NSF
P3	M	45.8	31.0	45.9	14.9	Y	L	Y	2	NSF
P4	F	48.8	3.0	49.0	46.0	Y	L	Y	1	SF
P5	M	28.2	23.0	28.6	5.6	Y	L	N	1	SF
P6	F	27.9	3.0	28.1	25.1	Y	L	N	1	SF
P7	F	45.8	7.0	46.0	39.0	Y	L	Y	1	SF
P8	F	31.4	13.0	31.6	18.6	Y	L	N	4	NSF
P9	F	41.9	9.0	42.5	33.5	Y	R	Y	1	SF
P10	M	44.9	12.0	45.1	33.1	Y	L	Y	1	SF
P11	F	47.6	11.0	47.7	36.7	Y	L	Y	5	NSF
P12	F	17.6	11.0	21.5	10.5	Y	R	N	1	SF
P13	F	30.8	16.0	31.0	15.0	Y	L	Y	1	SF
P14	M	26.3	7.0	26.4	19.4	N	L	Y	1	SF
P15	F	40.7	30.0	41.2	11.2	Y	L	N	5	NSF
P16	F	26.5	23.0	26.6	3.6	N	L	N	4	NSF
P17	F	29.0	2.5	30.2	27.7	N	R	N	1	SF
P18	M	32.1	17.0	32.5	15.5	N	L	Y	1	SF
P19	F	57.0	2.0	57.3	55.3	Y	R	Y	1	SF
P20	M	46.2	1.0	47.3	46.3	Y	L	Y	1	SF
P21	F	50.9	16.0	51.8	35.8	Y	R	Y	3	NSF
P22	M	33.7	20.0	35.2	15.2	Y	L	N	1	SF
P23	F	38.9	31.0	39.9	8.9	Y	L	N	2	NSF
P24	M	20.8	13.0	21.1	8.1	Y	L	Y	1	SF
P25	F	27.3	9.0	28.5	19.5	N	R	N	2	NSF
P26	F	20.5	17.0	21.6	4.6	N	R	N	1	SF
P27	F	18.5	2.0	19.1	17.1	Y	L	Y	2	NSF
P28	F	21.1	14.0	22.2	8.2	Y	R	Y	3	NSF
P29	M	51.0	35.0	51.3	16.3	Y	R	Y	3	NSF
P30	M	41.6	31.0	42.6	11.6	Y	R	Y	1	SF
P31	F	19.9	15.0	20.1	5.1	Y	R	N	1	SF
P32	F	40.5	10.0	41.9	31.9	Y	R	N	1	SF
P33	F	38.2	17.0	39.1	22.1	Y	R	Y	1	SF
P34	F	42.3	23.0	44.7	21.7	N	R	N	2	NSF
P35	F	46.7	7.0	48.0	41.0	Y	R	Y	1	SF
P36	F	66.8	50.0	68.1	18.1	Y	R	N	4	NSF
P37	M	44.9	14.0	47.7	33.7	N	R	N	4	NSF
P38	F	26.2	22.0	27.5	5.5	Y	R	N	1	SF
P39	F	19.8	13.0	20.3	7.3	Y	L	Y	1	SF
P40	M	29.6	4.5	31.7	27.2	Y	L	N	4	NSF
P41	F	26.8	0.9	27.3	26.4	Y	L	N	1	SF
P42	F	52.4	3.0	53.6	50.6	N	L	Y	3	NSF
P43	F	46.8	22.0	48.0	26.0	Y	R	Y	1	SF
P44	M	51.3	16.0	52.9	36.9	Y	L	Y	1	SF
P45	M	40.5	32.0	40.6	8.6	N	L	N	1	SF
P46	F	30.3	1.5	31.7	30.2	N	R	Y	1	SF
P47	F	43.5	19.0	44.6	25.6	N	R	N	3	NSF
P48	F	53.4	14.0	54.1	40.1	Y	R	Y	4	NSF
P49	F	37.9	34.0	38.6	4.6	Y	L	Y	2	NSF
P50	M	32.6	22.0	33.9	11.9	Y	L	N	1	SF
P51	F	31.2	10.0	32.5	22.5	N	R	N	1	SF
P52	F	24.8	7.0	25.3	18.3	Y	R	N	1	SF
P53	F	53.2	3.0	54.4	51.4	Y	R	Y	3	NSF
P54	M	38.2	2.0	39.1	37.1	Y	L	Y	1	SF
P55	M	38.9	8.0	40.2	32.2	Y	L	Y	2	NSF
P56	F	45.3	18.0	46.9	28.9	Y	R	N	4	NSF
P57	F	26.0	22.0	27.1	5.1	N	R	N	1	SF
P58	M	29.5	27.0	30.5	3.5	N	R	N	1	SF
P59	F	27.4	17.0	29.2	12.2	Y	L	Y	1	SF
P60	F	47.3	12.0	48.2	36.2	Y	R	Y	1	SF
P61	F	22.6	15.0	23.3	8.3	Y	R	N	1	SF
P62	F	29.8	7.0	30.6	23.6	N	R	Y	4	NSF

Subject	Sex	Age at dMRI (years)	Age at epilepsy onset (years)	Age at surgery (years)	Epilepsy duration (years)	History of FBTCS	Side of surgery	Hippocampal Sclerosis	ILAE outcome in 2 years	Surgical outcome
P63	F	40.9	21.0	42.2	21.2	N	L	Y	4	NSF
P64	F	43.1	38.0	43.2	5.2	N	L	N	1	SF
P65	F	45.0	15.0	45.3	30.3	N	L	Y	4	NSF
P66	M	41.8	25.0	42.7	17.7	Y	R	Y	3	NSF
P67	F	23.6	0.7	25.0	24.3	Y	R	N	1	SF
P68	M	52.0	0.0	52.9	52.9	Y	L	Y	1	SF
P69	M	24.7	13.0	26.2	13.2	Y	R	N	4	NSF
P70	M	19.1	11.0	20.6	9.6	Y	R	Y	1	SF
P71	M	53.5	43.0	54.6	11.6	Y	L	N	5	NSF
P72	M	59.0	5.0	60.2	55.2	Y	L	Y	1	SF
P73	F	44.5	35.0	45.3	10.3	Y	R	Y	4	NSF
P74	M	40.3	14.0	45.9	31.9	Y	R	N	1	SF
P75	M	43.8	0.8	46.4	45.7	Y	R	Y	3	NSF
P76	F	59.8	24.0	60.9	36.9	Y	L	N	1	SF
P77	M	36.3	13.0	37.0	24.0	Y	L	Y	3	NSF
P78	M	56.4	11.0	57.9	46.9	Y	L	Y	1	SF
P79	M	45.5	6.0	47.1	41.1	N	R	N	1	SF
P80	M	38.9	26.0	40.6	14.6	N	L	Y	5	NSF
P81	M	30.2	8.0	35.4	27.4	N	L	Y	1	SF
P82	F	33.2	22.0	33.4	11.4	Y	L	Y	2	NSF
P83	F	46.3	19.0	51.2	32.2	Y	L	N	3	NSF
C1	M	37.0								
C2	F	46.0								
C3	F	40.0								
C4	F	25.0								
C5	F	19.0								
C6	M	26.0								
C7	M	21.0								
C8	F	47.0								
C9	F	52.0								
C10	F	37.0								
C11	M	25.0								
C12	F	37.0								
C13	M	42.0								
C14	M	53.0								
C15	M	30.0								
C16	M	58.0								
C17	M	49.0								
C18	F	22.0								
C19	F	30.0								
C20	F	49.0								
C21	F	50.0								
C22	F	24.0								
C23	M	49.0								
C24	M	39.0								
C25	F	64.0								
C26	M	31.0								
C27	F	41.0								
C28	F	23.0								
C29	F	26.0								

Abbreviations: Female (F), Male (M), Left hemisphere (L), Right hemisphere (R), Yes (Y), No (N).
Surgical outcomes are defined as seizure free (SF) or not seizure free (NSF) based on ILAE outcomes recorded in two years after the surgery.
Patients who remained ILAE 1 in both year 1 and year 2 after surgery were deemed seizure free or else not seizure free.
